# Supplementary material for: Life-On-Hold: Lanthanoids Rapidly Induce a Reversible Ametabolic State in Mammalian Cells
Source: Biology (Basel). 2021 Jun 30;10(7):607. doi: 10.3390/biology10070607 (PMC8301128; doi:10.3390/biology10070607)
Supplement: Supplementary file 1 [file biology-10-00607-s001.zip › biology-1179452-Supplementary Materials_AS.pdf]

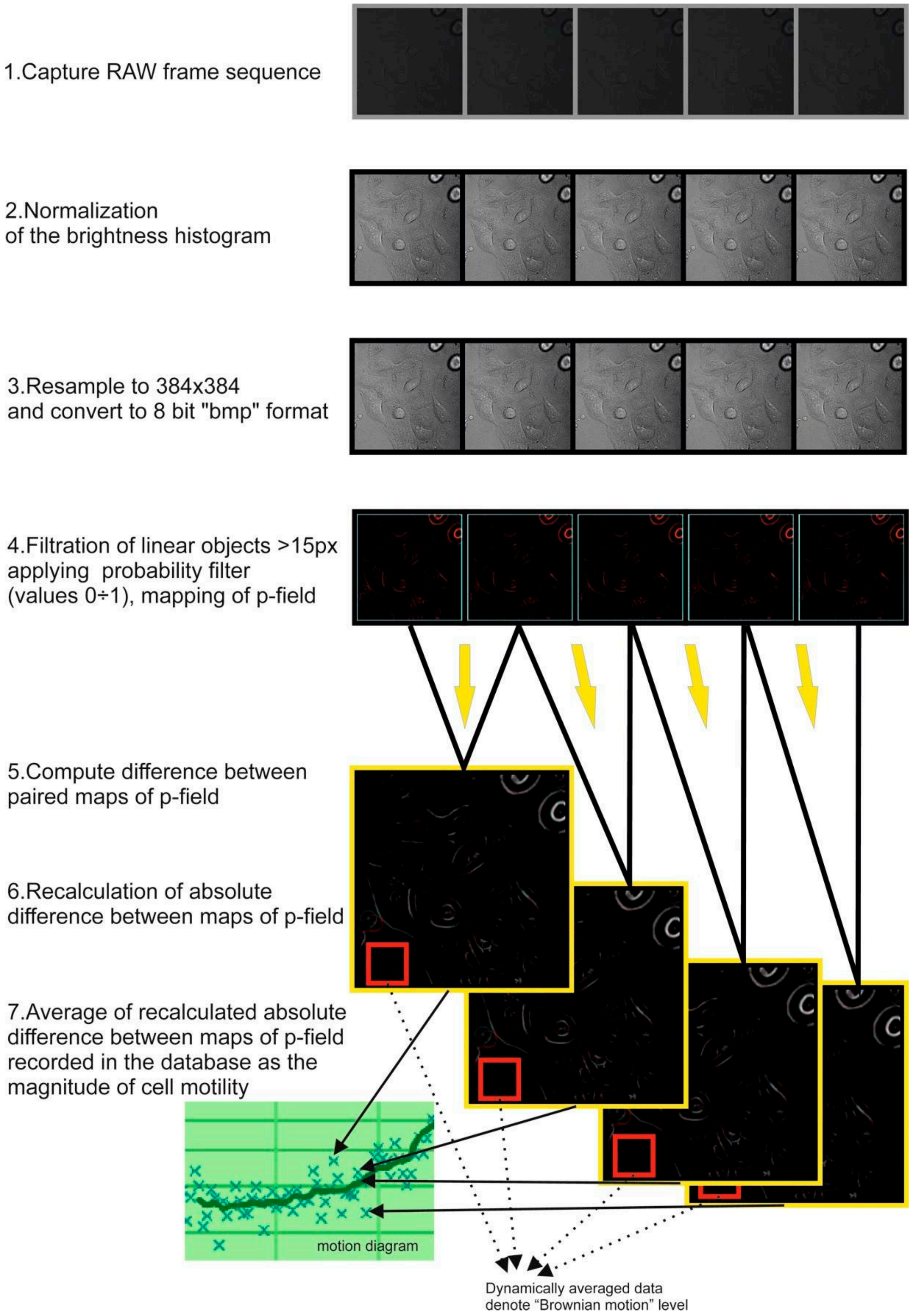

Figure S1. Image processing algorithm.

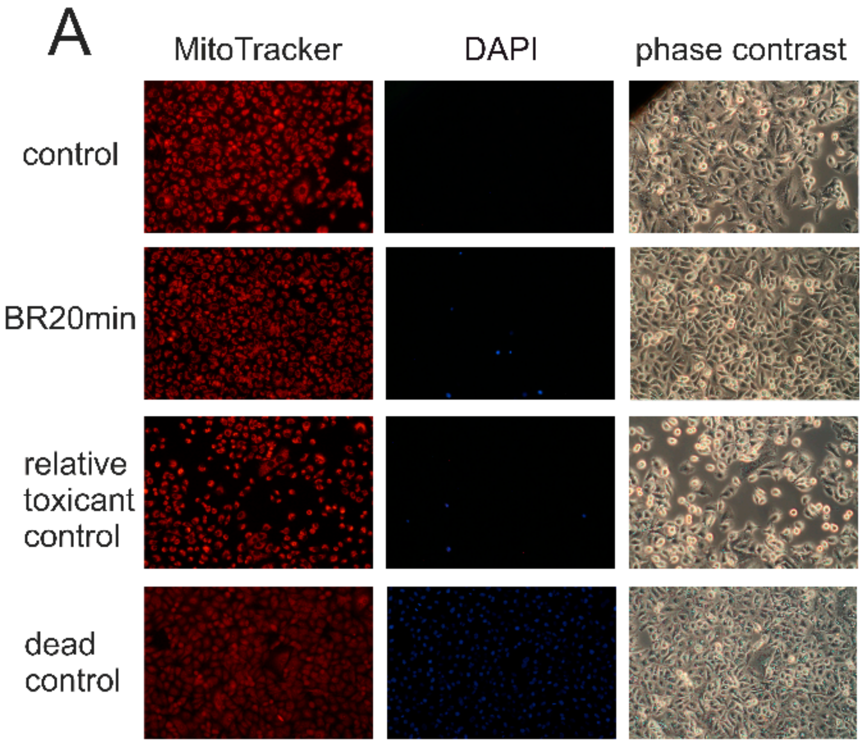
A
MitoTracker
DAPI
phase contrast
control
BR20min
relative toxicant control
dead control

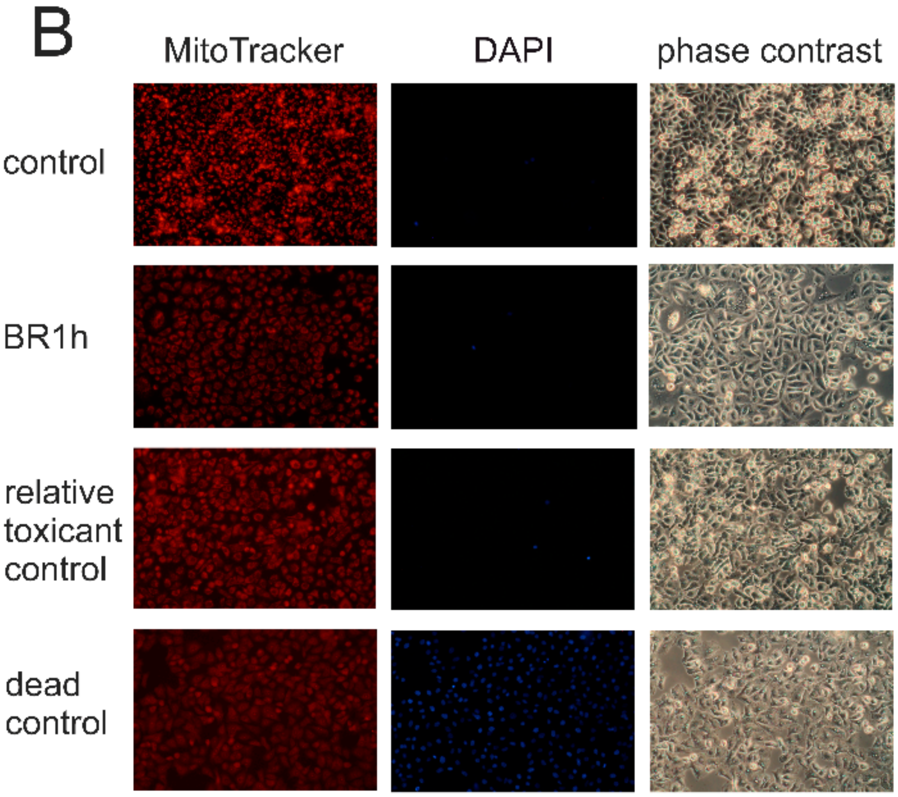
B
MitoTracker
DAPI
phase contrast
control
BR1h
relative toxicant control
dead control

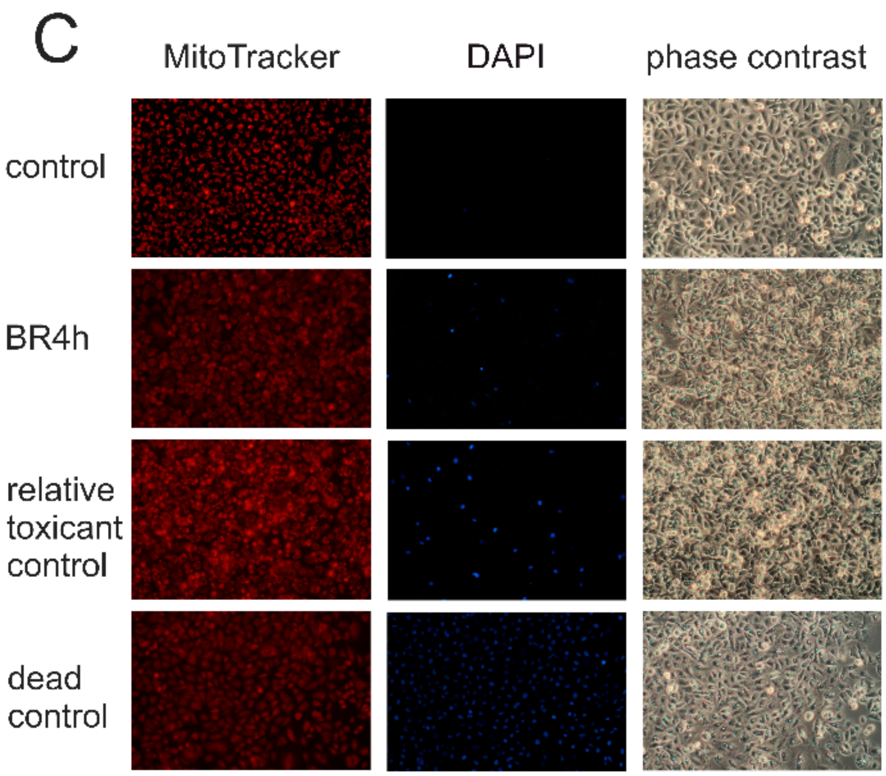

Figure S2. Epifluorescence and phase contrast view of a549 cell culture after lathanoid treatment and in control conditions after 20 min of exposition (**A**) 1h of exposition (**B**) and 4h of exposition (**C**). Mitotracker and DAPI staining.

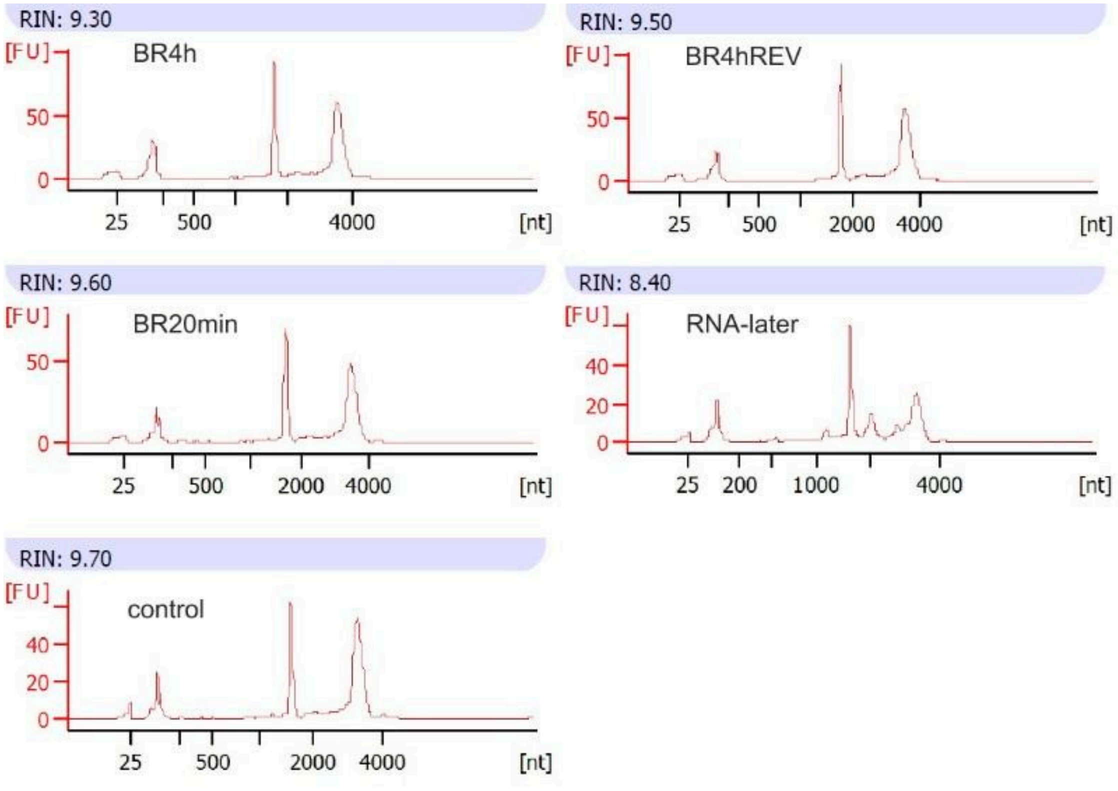

Figure S3. RNA stability in the cells after extended lathanoid treatment, RNAlater treatment and in the control untreated cells.
